# Theranostic in GLP-1R molecular imaging: challenges and emerging opportunities

**DOI:** 10.3389/fmolb.2023.1210347

**Published:** 2023-09-15

**Authors:** Yang Xie, Yudi Wang, Wenjie Pei, Yue Chen

**Affiliations:** ^1^ Department of Nuclear Medicine, The Affiliated Hospital of Southwest Medical University, Luzhou, Sichuan, China; ^2^ Nuclear Medicine and Molecular Imaging Key Laboratory of Sichuan Province, Luzhou, Sichuan, China; ^3^ Academician (Expert) Workstation of Sichuan Province, Luzhou, Sichuan, China

**Keywords:** GLP-1r, molecular imaging, theranostics, challenges, opportunities

## Abstract

Theranostic in nuclear medicine combines diagnostic imaging and internal irradiation therapy using different therapeutic nuclear probes for visual diagnosis and precise treatment. GLP-1R is a popular receptor target in endocrine diseases, non-alcoholic steatohepatitis, tumors, and other areas. Likewise, it has also made breakthroughs in the development of molecular imaging. It was recognized that GLP-1R imaging originated from the study of insulinoma and afterwards was expanded in application including islet transplantation, pancreatic β-cell mass measurement, and ATP-dependent potassium channel-related endocrine diseases. Fortunately, GLP-1R molecular imaging has been involved in ischemic cardiomyocytes and neurodegenerative diseases. These signs illustrate the power of GLP-1R molecular imaging in the development of medicine. However, it is still limited to imaging diagnosis research in the current molecular imaging environment. The lack of molecular-targeted therapeutics related report hinders its radiology theranostic. In this article, the current research status, challenges, and emerging opportunities for GLP-1R molecular imaging are discussed in order to open a new path for theranostics and to promote the evolution of molecular medicine.

## 1 Introduction

**FIGURE 1:**
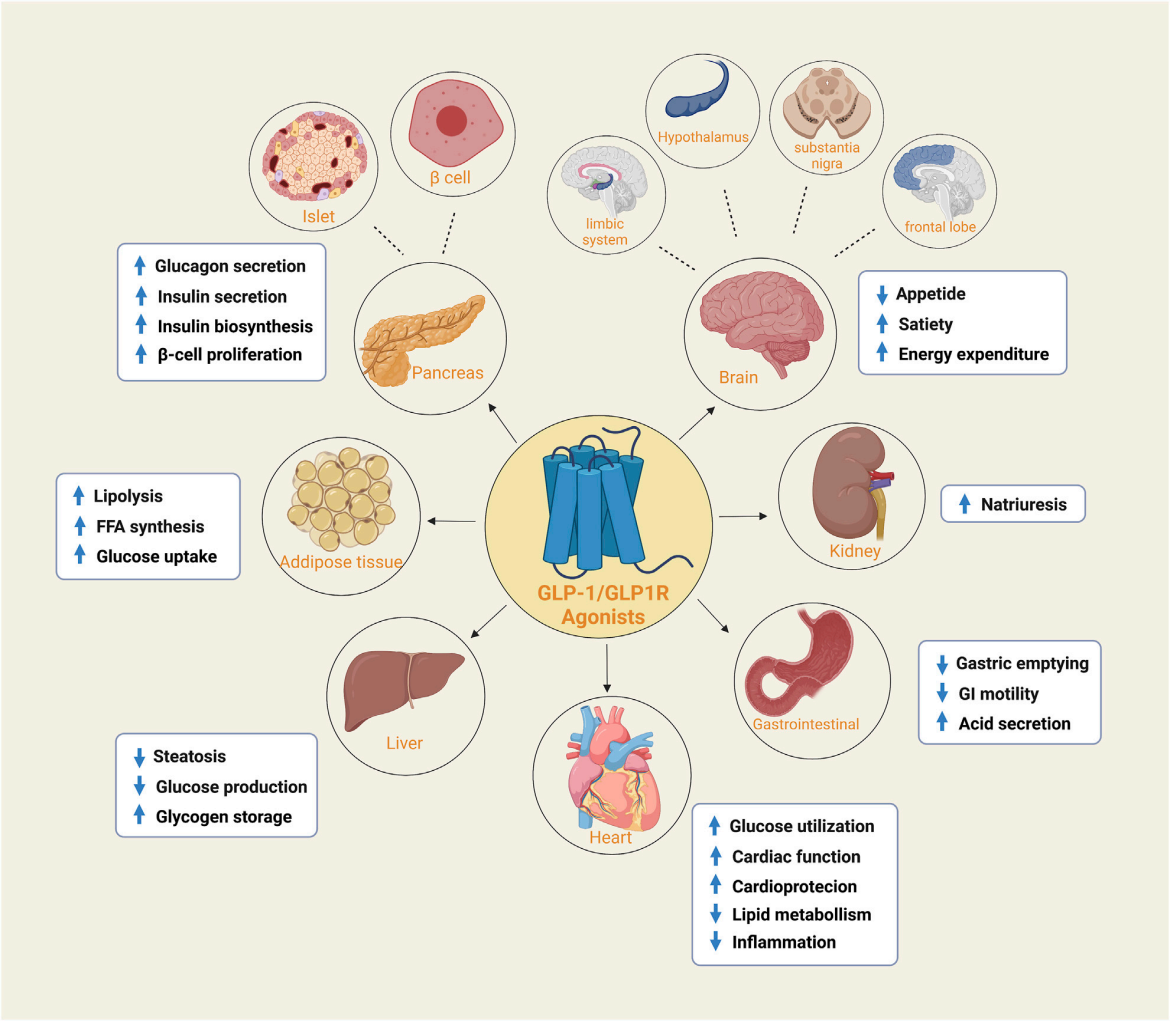
The expression of GLP-1/GLP-1R agonists in different organs and tissues. GLP1R is widely distributed and is expressed in both islets and beta cells of the pancreas, adipose tissue, heart, liver, kidney and gastroduodenum. It is also found in the frontal lobe, substantia nigra, hypothalamus and limbic system of the brain. They perform their respective functions in different tissue sites.

GLP-1R belongs to the subfamily of G protein-coupled receptors (Holst, 2007). It is widely distributed in pancreatic islets, the gastrointestinal tract, the heart, kidney, liver, brain, and other tissue organs (Pabreja et al., 2014). Various physiological functions can perform when activated by GLP-1 or synthetic agonists. In addition to its principal activity in islet cells, ghrelin inhibits gastric secretion and gastrointestinal motility, delays gastric emptying, increases satiety and decreases food intake in gastrointestinal tissues (Brubaker and Drucker, 2002). Similarly, GLP-1R protects nerve cells, fights loss of appetite, and enhances memory. In the cardiovascular aspect, it can improve cardiovascular function and reduce inflammation (Laviola et al., 2012) (Figure 1). Obviously, such a powerful physiological function and general distribution characteristics make it a popular receptor target in recent years.

GLP1, the natural ligand of GLP-1R, is rapidly degraded and inactivated by dipeptidyl peptidase IV (DPP-IV) *in vivo*. Based on this, the advent of GLP-1 analog opens a new direction for molecular imaging. Except the GLP1 analogue exendin-3 and its antagonist exendin-(9–39) are involved in GLP-1R molecular imaging studies as GLP-1 ligands. The most successful and widely used ligand for current GLP1R molecular imaging is still exendin-4. As the first generation GLP1 analog, it is a 39-amino acid peptide structure, which was discovered and extracted from Gila monster saliva and shared 53% identity with human GLP1 (Joosten et al., 2018). Meanwhile, it has a longer half-life (9.57 h) than GLP1 (about 2 min) because it lacks a specific site for DPP-IV hydrolase and is resistant to the rapid enzymatic digestion of DPP-IV (Zhang and Chen, 2012). With this natural advantage, it has become the core probe for GLP-1R molecular imaging.

**FIGURE 2:**
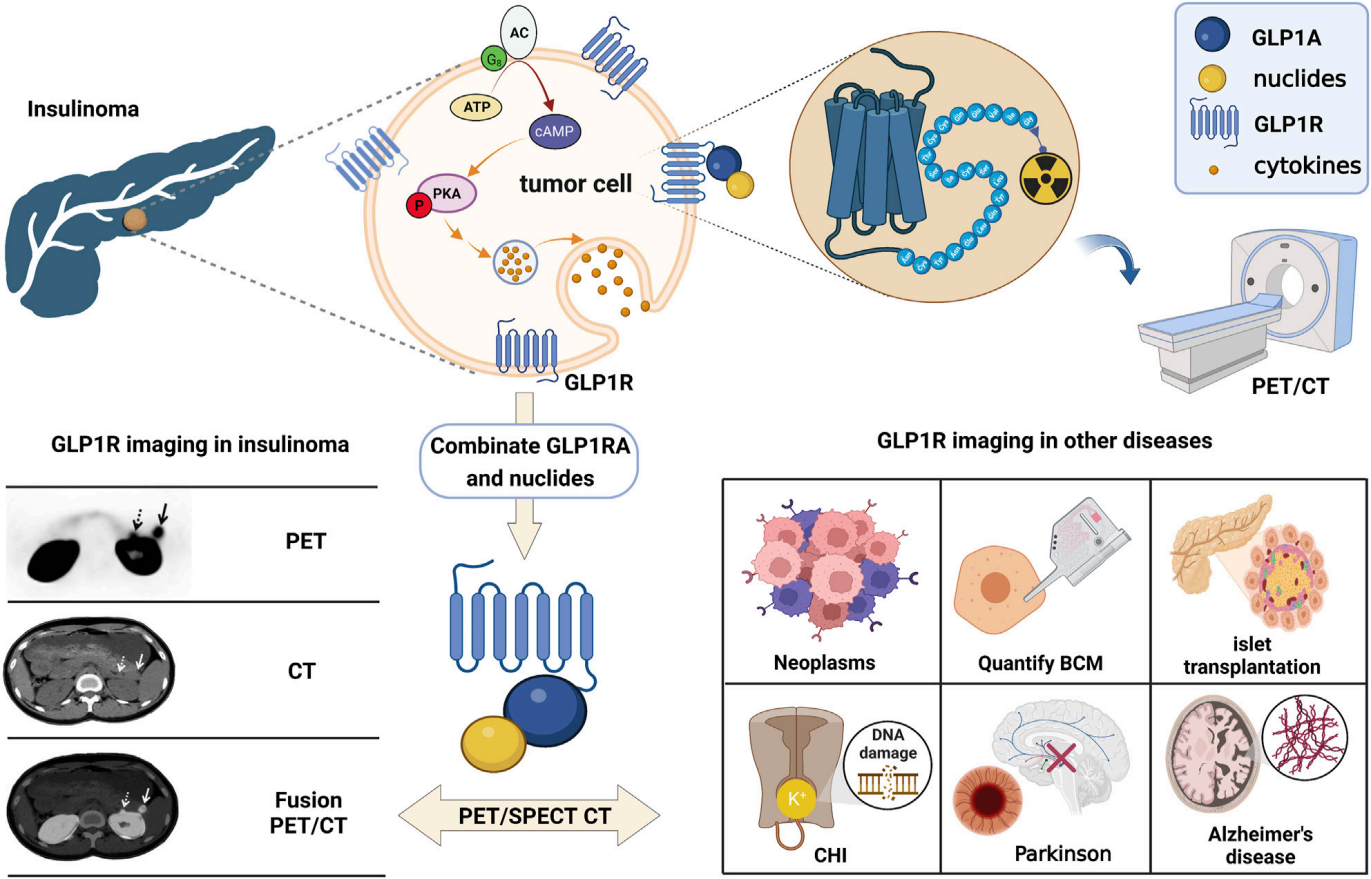
The schematic of GLP-1R molecular imaging. GLP1 analogues can bind competitively with GLP1 to GLP-1R targets and achieve GLP-1R molecular imaging by labelling with different radionuclides. Many studies of GLP-1R molecular imaging have been performed in tumours such as insulinoma, medullary thyroid cancer, myocardial ischaemia, islet transplantation, pancreatic beta-cell mass monitoring, congenital hyperinsulinemia and neurodegenerative diseases.

In the initial exploration of molecular imaging of GLP-1R, Korner (Korner et al., 2007) first labeled the radionuclide 125I on exendin-4. Excitingly, the biodistribution results demonstrated that the receptor is highly expressed in insulinoma, gastrinoma, pheochromocytoma, and medullary thyroid carcinoma (MTC). In addition, intracranial tumors such as meningiomas and astrocytomas have upregulated receptor manifestations. Among those neoplasms, the highest expression in benign insulinomas is consistent with Reubi’s autoradiography study (Reubi and Waser, 2003). Thus, the publication of these two findings were promoted directly with the development of molecular imaging on insulinoma. Although exendin-4, the most mature molecular probe for GLP-1R imaging, has been successful in insulinoma studies. We have aware that dependence on this tracer is currently the biggest challenge. The advancement of more comprehensive GLP-1R molecular imaging agents will also become an emerging opportunity. Fortunately, in recent years, research on it has gradually expanded to other fields such as medullary thyroid cancer, myocardial ischaemia, islet transplantation, islet beta cell mass monitoring, congenital hyperinsulinemia and neurodegenerative diseases (Figure 2). This article reviews the past and present development in GLP-1R molecular imaging. It puts forward the challenges encountered at this stage and new opportunities in the future, aiming to lay a solid theoretical basis for long-term theranostics.

## 2 GLP-1R molecular imaging in insulinoma

Even before the discovery of exendin-4, its homolog exendin-3 was successfully synthesized by 125I labeling and utilized in two models of insulinoma (NEDH rats and RINm 5F cells) (Gotthardt et al., 2002), which opened the chapter of GLP-1R imaging for 21 years. During this period, molecular imaging in insulinomas has matured, thanks to the exploration of exendin-4. It acts as a double-edged sword, pushing GLP-1R forward while relying on it so that all current molecular imaging studies of insulinoma focus on exendin-4 and its derivatives. This section summarizes the previous 21 years of GLP-1R molecular imaging in insulinoma. Insulinoma is the most common cause of endogenous hyperinsulinemic hypoglycemia (EHH) (Mori et al., 2020). As a result, detecting and precisely localizing the lesion prior to surgery is essential for surgical treatment (Falconi et al., 2016). However, the sensitivity of conventional imaging techniques for insulinoma is low, and the imaging is blurred. These factors affect the detection rate of lesions. Therefore, the molecular imaging study of GLP-1R was carried out.

### 2.1 Preclinical study of GLP-1 receptor molecular imaging in insulinoma

Initially (Wild et al., 2006), Wild was the first to successfully visualize insulinomas in preclinical studies utilizing Ahx as a linker between DTPA and peptide by c-terminal extension of the Lys side chain coupling of exendin-4 (Lys40NH2 modifies Exendin-4), a landmark for GLP-1R molecular visualization. Wicki et al. (2007) later observed a 94% reduction in tumor volume within 8 days after injecting 28 MBq of radioactive peptide under the same molecular tracer. Such positive results raise expectations for driving molecular imaging in the theranostic of insulinoma. Nonetheless, the 111In is costly and has a significant radiation burden on patients. Therefore, an alternative radionuclide for SPECT/CT imaging, 99mTc, appeared in comparison research with 111In at Wild et al. (2010). The synthesized molecular compound that used exendin-4 as a substrate also demonstrated an ideal tumor background ratio and insulinoma sensitivity, making it an ideal choice for GLP-1R molecular imaging.

**FIGURE 3:**
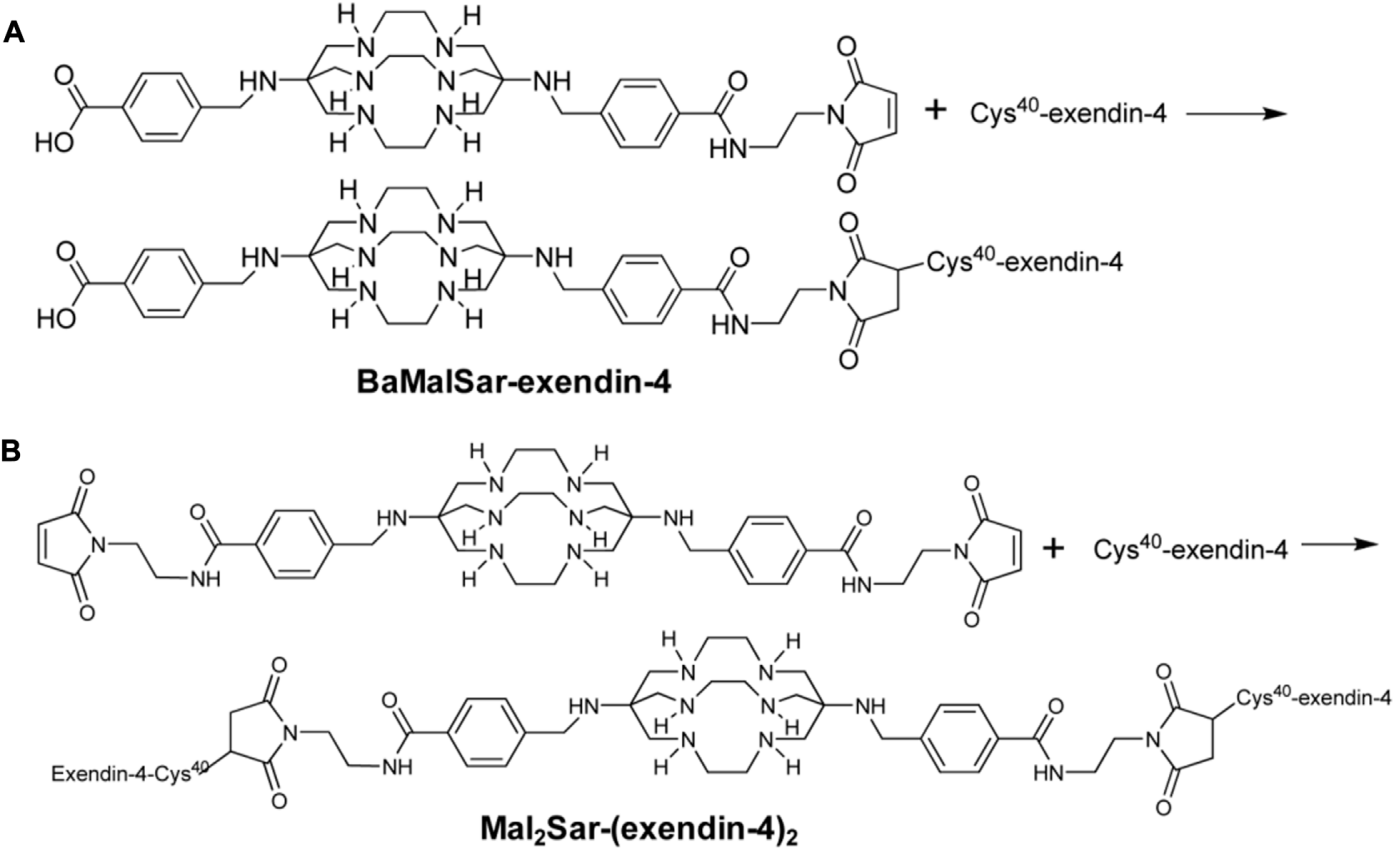
Synthesis scheme of monomeric and dimeric exendin-4 analogs. Two novel peptide structures BaMalSar-exendin-4 **(A)** and Mal2Sar-(exendin-4)_2_ **(B)** were respectively prepared by coupling the ligand Cys-exendin-4 with BaMalSar and Mal2Sar, in over 90% yield.

Regrettably, the above studies were all SPECT/CT imaging of nuclide markers until Wu’s two results (Wu et al., 2011; Wu et al., 2014) on 64Cu-exendin-4 PET/CT molecular imaging of insulinomas opened the window to new horizons of GLP-1R molecular imaging again. The ligands Cys-exendin-4 were respectively added to phosphate buffers containing BaMalSar and Mal_2_Sar at pH 7.0, and the coupling products BaMalSar-exendin-4 and Mal2Sar-(exendin-4)2 were purified by HPLC with stirring at room temperature in yields greater than 90% (Figure 3). Not only did they visualize insulinomas using this molecular imaging, but also successfully visualized portal islet transplantation models. However, negative findings could also reveal that the average effective dose of 64Cu was nearly ten times higher than that of 68Ga-exendin-4, which significantly increased the radiation dose and limited its application and translation. It suggests that 68Ga may be a nuclide probe with great potential for molecular imaging of GLP-1R.

Before beginning insulinoma research with 68Ga-exendin-4, several results (Kiesewetter et al., 2012a; Kiesewetter et al., 2012b; Keliher et al., 2012; Wu et al., 2013; Yue et al., 2013; Xu et al., 2015; Li et al., 2020; Murakami et al., 2021a) with 18F indicated that 18F-exendin-4 is critical for developing molecular imaging. Surprisingly, in these 18F experiments, we saw one of the most significant drawbacks of current GLP-1R molecular imaging, namely, high renal uptake, which can lead to missed or misdiagnosed lesions located in the tail of the pancreas next to the kidney. Actually, the high renal radiation burden emerged early when Vegt et al. (2008) and Wild et al. (2010) reduced renal uptake by adding the albumin fragment FRALB, gelofusine, and polyglutamic acid infusion. However, the tedious and complicated preparation constrained subsequent development. In contrast, the 18F-exendin-4 study was performed by synthesizing new GLP-1R derivatives through diverse modifications of different amino acid structural sites of Exendin-4 to obtain a lower renal load developer. We have seen that successfully preparing different modified derivatives, including micron and nanomaterials [FBEM (Kiesewetter et al., 2012a), TCO (Keliher et al., 2012), TTCO (Wu et al., 2013), FPenM (Yue et al., 2013), and PEG (Li et al., 2020; Murakami et al., 2021a)], can reduce renal uptake to a certain extent yet the process is still complicated. It is possible that finding new analogs of GLP-1 with higher homology and a nephroprotective native structure will be a target for future molecular imaging works. Moreover, perhaps the second-generation GLP-1 analog semaglutide will be a new option.

**FIGURE 4:**
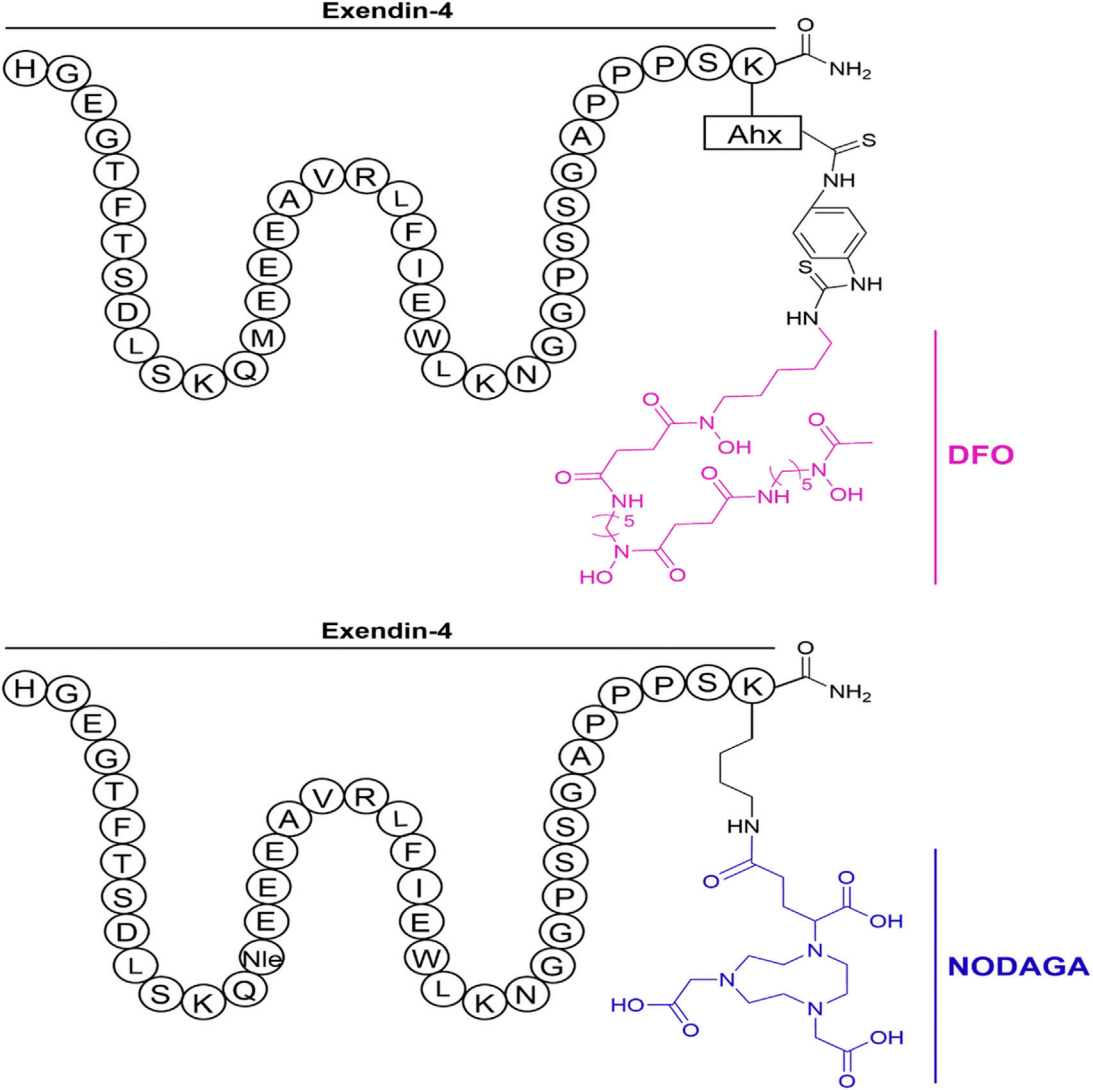
Schematic representation of the peptides investigated in the study. The top shows Ex4DFO and the bottom shows Ex4NOD. The chelators are depicted without coordinated radiometal.

Subsequently, the six preclinical studies (Brom et al., 2010; Sel et al., 2014; Bauman et al., 2015; Kaeppeli et al., 2019; Zhang et al., 2019) of 68Ga-exendin-4 held promise for further clinical translation and laid a solid foundation for its future unassailable position in insulinoma molecular imaging. In addition to the most common DOTA, using different chelators, including NOTA, NODAGA, DO3A, and DFO, has also compensated for the lack of singularity of other nuclide markers in the past. It was the first time in the Kaeppeli study (Kaeppeli et al., 2019) that exendin-4 was modified at position 14, where methionine (Met) was replaced by norleucine (Nle), to improve oxidative stability during labelling. And coupled to NODAGA to form [Nle14-Lys40-(NODAGA)NH2]exendin-4, referred to as Ex4NOD. A comparative study was carried out with Ex4DFO coupled to desferrioxamine DFO (Figure 4 of the two chemical structures). In particular, two comparative studies by Brom et al. (2010) and Wild et al. (2010) have shown that GLP-1R PET/CT molecular imaging has a better sensitivity and tumor background ratio for insulinoma than SPECT/CT.

We have summarized the previous preclinical studies of GLP1-R molecular imaging in insulinoma (Table 1) and discovered that from 111In in the early days of exploration to the now mature 68Ga. Even 89Zr (Bauman et al., 2015), a long half-life nucleophile, has been added to the list. It opens up more options for clinical research to be reviewed. Even so, the molecular probe for GLP-1R is still exendin-4. Such limitations and its high renal load are currently a challenge in this field, and perhaps the integration of new GLP-1R analogs into targeted therapy will become a more worthwhile prospect.

**TABLE 1:** Summary of preclinical studies of GLP-1R molecular imaging in insulinoma.

Nuclide	Year/Author/Reference	Chelating agent	Compound	Animal model	Dose	Tumor uptake (%ID/g)	Renal uptake (%ID/g)	Pancreas uptake (%ID/g)	Block test tumor uptake (% ID/g)	SUV and diamete of tumor
^111^IN										
	2006 Wild (12)	DTPA	[Lys40(AhxDTPA-111In)NH2]exendin-4	Rip1Tag2 mice	37 Mbq	287.37 ± 62.37	208.77 ± 34.68	21.13 ± 7.35	7.28 ± 3.50	287.37 ± 62.37 1-3.2 mm
	2007 Wicki (13)	DTPA	[Lys40 (AhxDTPA-111In)NH2]exendin-4	Rip1Tag2 mice	37 Mbq	307 ± 38	190 ± 11	10.1 ± 2.2	NA	307 ± 38
	2010 Wild (14)	DOTA	[Lys40(AhxDOTA-111In)NH_2_]exendin-4	Rip1Tag2 mice	37 Mbq	213 ± 75	243 ± 17	17.8 ± 3.9	9.35 ± 4.18	213 ± 75
	2010 Broom (29)	DTPA	[Lys40 (AhxDTPA-111In)NH_2_]exendin-4	INS-1 xenograft models BALB/c mice	3.7 MBq	36.3 ± 8.0	140.5 ± 17.7	18.9 ± 2.3	1.0 ± 0.4	25.4 ± 7.2 2–5 mm
^99^mTc										
	2010 Wild (14)	HYNIC	[Lys40 (Ahx-HYNIC-99mTc/EDDA)NH_2_]-Exendin-4	Rip1Tag2 mice	37 Mbq	93.1 ± 19.9	60 ± 12	7.4 ± 2.2	5.45 ± 0.43	93.1 ± 19.9 1–3.2 mm
^64^CU										
	2011 Wu (15)	DO3A	64Cu-DO3A-VSCys40-Exendin-4	INS-1 xenograft models NOD/SCID mice	3.7–7.4 MBq	14.2 ± 2.6	79.7 ± 6.83	8.2 ± 1.7	2.7 ± 2.0	14.2 ± 2.6
	2014 Wu (16)	BaMalSar	64Cu-BaMalSar-exendin-4	INS-1 xenograft models NOD/SCID mice	74 MBq	11.41 ± 2.91	124.68 ± 27.38	NA	1.41 ± 0.08	11.41 ± 2.91
	2014 Wu (16)	Mal2Sar	64Cu-Mal2Sar-(exendin-4)2	INS-1 xenograft models NOD/SCID mice	74 MBq	23.29 ± 5.35	82.19 ± 7.32	NA	1.49 ± 0.11	23.29 ± 5.35
^18^F										
	2012 Kiesewetter (17)	FBEM	[18F]FBEM [Cys40]-exendin-4	INS-1 and MDA-MB-435 xenograft models	3.7 MBq	7.20 ± 1.26	11.23 ± 2.26	3.74 ± 0.84	0.79 ± 0.08	25.25 ± 3.39
	2012 Kiesewetter (19)	NOTA	18F]AlF-NOTA-MAL-cys40-exendin-4	INS-1 xenograft models	3.7 MBq	17.9 ± 1.4	74.68 ± 6.20	2.28 ± 0.35	0.18 ± 0.02	14.6 ± 1.3
	2012 Keliher (21)	TCO	18F-E4Tz12 7	NIT-1, WTRT2, 916-1 xenograft models (C57BL/6)	3.7 MBq	2.5% (916-1) 2.0% (WTRT2) 0.7% (nit1)	17.8% ± 0.6%	1.2 ± 0.2	916-1: 82% reduction WTRT-2: 54% reduction it −1: 62% reduction	2.5% (916-1) 2.0% (WTRT2) 0.7% (nit1)
	2013 Yue (18)	FPenM	[18F]FPenM-[cys40]-exendin-4	INS-1 xenograft models BALB/c mice	3.7 MBq	20.32 ± 4.36	11.30 ± 2.41	7.32 ± 0.63	2.65 ± 0.63	20.32 ± 4.36
	2013 Wu (22)	TTCO	18F-TTCO-Cys40-exendin-4	INS-1 xenograft models NOD/SCID mice	3.7 MBq	16.03 ± 1.1	22.4 ± 5.3	NA	1.84 ± 0.77	16.03 ± 1.1
	2015 Xu (20)	NOTA	[18F]AlFNOTA-MAL-Cys39-exendin-4	INS-1 xenograft models BALB/c mice	3.7 MBq	7.11 ± 0.65	85.32 ± 5.89	1.84 ± 0.17	0.37 ± 0.01	7.74 ± 0.87
	2020 Li (23)	PEG4-TTCO	[18F] PTTCO-Cys40-Exendin-4	INS-1 xenograft models NOD/SCID mice	1.85 MBq	9.32 ± 0.75	17.67 ± 1.47	3.63 ± 0.11	3.97±0.88	9.32 ± 0.75
	2021 Murakami (24)	PEG	[18F] FB(ePEG12)12-exendin-4	INS-1 xenograft models BALB/c slc-nu/nu mice (Right thigh were implanted)	10 Mbq	37.3 ± 4.5	Tumor kidney ratio 3.30 ± 0.36	Tumor pancreas ratio 2.39 ± 0.39	NA	28.6 ± 4.4 11.0 ± 2.1 mm
	2021 Murakami (24)	PEG	[18F] FB(ePEG12)12-exendin-4	INS-1 xenograft models BALB/c slc-nu/nu mice (pancreas were implanted)	10 Mbq	37.7 ± 4.4	Tumor kidney ratio 3.44 ± 1.00	Tumor pancreas ratio 2.71 ± 0.27	NA	37.1 ± 0.4 7.6 ± 1.2 mm
^68^Ga										
	2010 Wild (14)	DOTA	[Lys40 (Ahx-DOTA-68Ga)NH2]-exendin-4	Rip1Tag2 mice	130 Kbq	205 ± 59	202 ± 34	13.5 ± 1.0	4.82	205 ± 59 2.3 mm
	2014 Selvaraju (26)	DO3A	[68Ga]Ga-DO3A-VS-Cys40-Exendin-4	INS-1 xenograft models BALB/c mice	2.5 μg/kg	30.06 ± 11.21	96.2 ± 6.8	12.5 ± 5.8	7.6 ± 3.2	44.8 ± 14.5
	2015 Bauman (30)	DFO	[Lys40-(AHX-DFO-68Ga)NH2]exendin-4	rin-m5f xenograft models Foxn1NU nude mice	100 KBq	32.5 ± 8.3	152.8 ± 25.4	11.3 ± 5.6	3.9 ± 0.9 (88%)	32.5 ± 8.3
	2019 Zhang (27)	NOTA	[68Ga]Ga-NOTA-MAL-Cys39-exendin-4	INS-1 xenograft models BALB/c mice	3.7 MBq	12.87 ± 0.89	56.86 ± 5.58	1.99 ± 0.22	1.05 ± 0.41	12.20 ± 0.89
	2019 Kaeppeli (28)	NODAGA	[68Ga]Ga-Ex4NOD	CHL xenograft models CD1 nu/nu mice	100 KBq	46.18 ± 13.72	105.27 ± 29.56	10.05 ± 1.25	4.13 ± 1.22	46.18 ± 13.72
	2019 Kaeppeli (28)	DFO	[68Ga]Ga-Ex4DFO	CHL xenograft models CD1 nu/nu mice	100 KBq	15.1 ± 2.67	524.73 ± 97.35	26.17 ± 5.48	5.89 ± 1.43	15.1 ± 2.67
	2010 Broom (29)	DOTA	[Lys40 (Ahx-DOTA-68Ga)NH_2_]-exendin-3	INS-1 xenograft models BALB/c mice	3.7 MBq	36.3 ± 8.0	140.5 ± 17.7	18.9 ± 2.3	1.0 ± 0.4	25.4 ± 7.2 2–5 mm
^89^Zr										
	2015 Bauman (30)	DFO	[Lys40-(AHXDFO-89Zr)NH_2_]exendin-4	rin-m5f xenograft models Foxn1NU nude mice	100 KBq	13.5 ± 0.8	216.9 ± 56.2	7.2 ± 2.3	2.6 ± 0.2 (81%)	13.5 ± 0.8

### 2.2 Clinical study of GLP-1 receptor molecular imaging in insulinoma

The theranostic of molecular medicine is the latest trend. Furthermore, the success of clinical translation more or less determines the future of molecular imaging of GLP-1R in insulinoma. We have likewise summarized all previous studies on insulinomas (Table 2). Nucleophiles18F, 64Cu, and 89Zr have restricted their clinical application due to some of the limitations mentioned in the previous section. Wild combines GLP-1R molecular imaging with the clinic for the first time (Wild et al., 2008; Christ et al., 2009; Wild et al., 2011), 2 years after its landmark 2006 preclinical study was published. Despite the small number of insulinoma patients included in the two clinical studies, 111In-DTPA-exendin-4 SEPCT/CT successfully detected lesions compared to the weak sensitivity of conventional imaging pairs. It opens new doors for molecular imaging of insulinomas. Subsequent SEPCT/CT molecular imaging investigations of 111In and 99mTc-labelled exendin-4 have followed (Sowa-Staszczak et al., 2013; Antwi et al., 2015; Senica et al., 2020). However, the most representative of these undoubtedly belongs to two large sample-size clinical trials by Christ in 2013 (Christ et al., 2013) and Sowa-Staszczak in 2016 (Sowa-Staszczak et al., 2016). Christ performed 111In-DTPA-exendin-4 molecular imaging in 20 of the 30 patients with EHH who were included to detect insulinomas and successfully detected 19 with a sensitivity of 95%, in contrast to 44% with conventional imaging. Such results are consistent with Sowa-Staszczak. The latter used 99mTC-HYNIC-exendin-4 in all 18 patients with insulinoma, significantly higher than the detection rate of 6/18 (33.3%) with conventional imaging. Such promising results are encouraging, and it indicates that GLP1-R molecular imaging is gradually replacing conventional imaging as the non-invasive modality of choice for the accurate preoperative localization of insulinoma.

**TABLE 2:** Summary of clinical studies of GLP-1R molecular imaging in insulinoma.

Nuclide	Year/Author/Reference	Chelating agent	Compound	Dose	EHH	Pathological examination	Benign insulinoma	Malignant insulinoma	MEN-1	CHI	Positive of GLP-1R imaging (sensibility)	Positive of CT/MRI/EUS/SSTR imaging (sensibility)	Benign insulinoma by GLP-1R imaging (sensibility)
^111^IN													
	2008 Wild (31)	DTPA	[Lys40(AhxDTPA-111In)NH_2_]exendin-4	90 MBq	2	2	2/2	0	0	0	2/2 (100%)	0/2 (−)	2/2 (100%)
	2009 Christ (32)	DOTA	[Lys40(AhxDOTA-111In)NH_2_]exendin-4	82–97 MBq	6	6	6/6	0	0	0	6/6 (100%)	4/6 (66.7%)	6/6 (100%)
	2011 Wild (33)	DTPA	[Lys40(AhxDTPA-111In)NH_2_]exendin-4	108–136 MBq	11	11	0/11	11/11	0	0	4/8 (50%)	8/11 (72.7%)	None
	2013 Christ (37)	DTPA	[Lys40 (AhxDTPA-111In)NH_2_]exendin-4	80–128 MBq	30	25	20/25	2/25	2/25	3/5	23/25 (92%)	11/25 (44%)	19/20 (95%)
	2015 Antwi (36)	DOTA	[Lys40(AhxDOTA-111In)NH_2_]exendin-4	66–90 MBq	5	4	4/4	0	0	0	2/5 (40%)	1/5 (20%)	2/4 (50%)
^99^mTc													
	2013 Sowa-Staszczak (34)	HYNIC	[Lys40(Ahx-HYNIC-99mTc/EDDA)NH_2_]-Exendin-4	740 MBq	11	11	8/11	2/11	0	1/11	9/11 (81.8%)	4/8 (50%)	8/8 (100%)
	2016 Sowa-Staszczak (38)	HYNIC	[Lys40(Ahx-HYNIC-99mTc/EDDA)NH_2_]-Exendin-4	740 MBq	40	19	18/19	1/19	0	0	28/40 (70%)	6/18 (33.3%)	18/18 (100%)
	2020 Senica (35)	HYNIC	[Lys40(Ahx-HYNIC-99mTc/EDDA)NH_2_]-Exendin-4	740 MBq	8	8	8/8	0	0	0	8/8 (100%)	4/8 (50%)	8/8 (100%)
^68^Ga-DOTA													
	2015 Antwi (36)	DOTA	68Ga-DOTA-Exendin-4	76–97 MBq	5	4	4/4	0	0	0	5/5 (100%)	1/5 (20%)	4/4 (100%)
	2019 Sood (40)	DOTA	68Ga-DOTA-Exendin-4	111 MBq	1	1	1/1	0	0	0	1/1 (100%)	0/1 (−)	1/1 (100%)
	2020 Michalski (42)	DOTA	68Ga-DOTA-Exendin-4	84 ± 21 MBq	10	7	7/7	0	0	2	8/10 (80%)	2/7 (28.6%)	7/7 (100%)
	2021 Das (41)	DOTA	68Ga-DOTA-Exendin-4	111 MBq	1	1	1/1	0	0	0	1/1 (100%)	0/1 (−)	1/1 (100%)
	2021 Zhang (43)	DOTA	68Ga-DOTA-Exendin-4	111 MBq	1	1	1/1	0	0	0	1/1 (100%)	0/1 (−)	1/1 (100%)
	2023 Qiu (39)	DOTA	68Ga-DOTA-Exendin-4	111 MBq	1	2	2/2	0	0	0	2/2 (100%)	1/2 (50%)	2/2 (100%)
^68^Ga-DO3A													
	2017 Velikyan (44)	DO3A	[68Ga]Ga-DO3A-VS-Cys40-Exendin-4	50 MBq	1	1	1/1	0	0	0	1/1 (100%)	0/1 (−)	1/1 (100%)
	2014 Eriksson (45)	DO3A	[68Ga]Ga-DO3A-VS-Cys40-Exendin-4	56 MBq	1	1	1/1	0	0	0	1/1 (100%)	0/1 (−)	1/1 (100%)
^68^Ga-NOTA													
	2015 Luo (46)	NOTA	68Ga-NOTA-exendin-4	51.8 MBq	1	1	1/1	0	0	0	1/1 (100%)	0/1 (−)	1/1 (100%)
	2016 Luo (47)	NOTA	68Ga-NOTA-exendin-4	51.8 MBq	1	1	1/1	0	0	0	1/1 (100%)	0/1 (−)	1/1 (100%)
	2017 Luo (49)	NOTA	68Ga-NOTA-exendin-4	18.5–185 MBq	52	43	43/43	0	0	0	44/45 (97.8%)	39/43 (90.7%)	42/43 (97.7%)
	2020 Warren (48)	NOTA	68Ga-NOTA-exendin-4	51.8 MBq	1	1	1/1	0	0	0	1/1 (100%)	0/1 (−)	1/1 (100%)
^68^Ga-NODAGA													
	2019 Boss (50)	NODAGA	68Ga-NODAGA-exendin-4	95–105 Mbq	34	29	29/29	0	0	0	29/34 (85.3)	20/30 (66.7%)	29/29 (100%)

**FIGURE 5:**
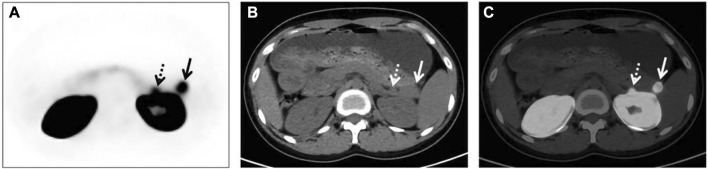
GLP-1R molecular imaging of double-primary insulinomas. Two insulinoma lesions in the tail of the pancreas, only one of which was detected in conventional imaging.68 Ga-DOTA-exendin-4 molecular imaging successfully detected both lesions and guided surgical resection. In the axial images [**(A)** PET, **(B)** CT, **(C)** fusion) in addition to the significantly higher uptake of the lesion in the tail of the pancreas, we see that GLP-1R imaging successfully detected the other lesion adjacent to the kidney, which was not detected in the MRI image due to the obscuration of the kidney. Thus, GLP-1R molecular imaging offers better advantages over conventional imaging in the preoperative precise localization of insulinomas.

Similarly, PET/CT studies, a representative of molecular imaging, have also revolved around 68Ga-exendin-4. Different chelator-targeted molecular probes have been successfully reported in several cases. Here, our team (Qiu et al., 2023) also successfully used 68Ga-DOTA-exendin-4 PET/CT to visualize a double lesion insulinoma in the pancreas’s tail, one of which was not detected on MRI (Figure 5). The clinical reports of Ga-exendin-4 are among the most diverse in terms of the chelating agents employed, and it has been an essential part of its success in clinical translation. Whether DOTA (Sood et al., 2019; Michalski et al., 2020; Das et al., 2021; Zhang et al., 2021), DO3A (Eriksson et al., 2014; Velikyan et al., 2017), or NOTA (Luo et al., 2015; Luo et al., 2016a; Warren et al., 2020) is reported on a case-by-case basis, the most convincing report and one that has driven the recognition of 68Ga-GLP-1R molecular imaging should be the publication of Luo’s seminal study in 2017 (Luo et al., 2016b). He prepared 68Ga-NOTA-MAL-cys40-exendin-4 by coupling NOTA-MAL with the ligand Cys40-exendin-4 and then successfully targeting 68Ga to the peptide at 100°C for 15 min. The results revealed that 68Ga-NOTA-exendin-4 PET/CT successfully detected lesions in 42 of 43 insulinoma patients, with a sensitivity of 97.7%, markedly higher than the 19.5% of 99mTc-HYNIC-TOC SPECT/CT. It is the first time that the two types of molecular imaging have been compared again in a clinical report, and PET/CT remains the obvious choice. Another study (Boss et al., 2019), also widely expected, showed a sensitivity of 85.3% for 68Ga-NODAGA-exendin-4 PET, greater than SSTR PET (54.5%) and conventional imaging (66.7%). At this point GLP-1R molecular imaging has entered the era of 68Ga-exendin-4, and their meaning here cannot be overstated.

Unfortunately, it has been greatly appreciated in molecular imaging as the most popular combination of 68Ga/177Lu for theranostics. The research is still vacant in insulinoma. As early as 2016 (Espes et al., 2016) [177Lu]-DO3A-VS-Cys40-Exendin-4 was used for the first time to successfully scan transplanted islets and quantify cell mass in an islet transplantation model. In 2019, Guleria et al. (2019) produced a molecule [177Lu]Lu-DOTA-Ahx-Lys40-Exendin-4 with high radiochemical purity. The biodistribution investigation revealed that the chemical initially accumulated in pancreatic tissue and was predominantly excreted by the kidney. Nevertheless, its substantial absorption in the kidney restricts its potential for further translation. The failure of the investigations above based on 177Lu-labeled exendin-4 to complete GLP-1R molecular imaging and targeted therapy in insulinoma models will present an emerging opportunity for our long-term research. It will also be the biggest obstacle to the current move towards GLP-1R molecular imaging into theranostic, and we need to pool our efforts to overcome this challenge.

## 3 Emerging opportunities for GLP-1R molecular imaging

Although most past studies have been limited to insulinomas, we tabulated radiolabeling options from all relevant studies (Table 3). It is easy to see that the preclinical studies cover a much wider choice of radionuclides, but the large radiation load of 64Cu, the stringent labelling conditions of 18F and the cumbersome preparation of these radionuclides, for example. These are all constraints to their clinical translation. The two long half-life radiolabels, 89Zr and 177Lu, have also failed to achieve clinical translation, even though they offer better therapeutic prospects. However, the greater renal burden of both and the limited stability of 89Zr due to the covalent bond breakage between chelator and protein have limited their potential use in GLP-1R imaging. Therefore both 111In and 99mTc in SPECT/CT and radionuclide 68Ga in PET/CT are better choices for GLP-1R radiolabelling. It also provides a guarantee and basis for future research in other areas of clinical translational research and diagnostic integration.

**TABLE 3:** List of GLP-1R molecular imaging radionuclides.

Nuclide	PET/SPECT	Half-life (T_1/2_)	Maximum radiation energy	Cyclotron/Reactor	Application	Limitations
^111^IN	SPECT	2.81 days	0.171 MeV	^111^Cd(p,n)^111^In	preclinical/clinical	costly and has a significant radiation burden on patients
^99m^Tc	SPECT	6.02 h	0.140 MeV	^100^Mo(p,2n)^99m^Tc	preclinical/clinical	SPECT imaging has a significantly lower resolution than PET imaging
^64^Cu	PET	12.7 h	β+ 0.655 MeV β- 0.573 MeV	^64^Ni(p,n)^64^Cu	preclinical	the radiation dose nearly ten times higher than that of ^68^Ga-exendin-4
^18^F	PET	110 min	β 0.634 MeV γ Vminh MeV	^18^O(p,n)^18^F	preclinical	Harsh marking conditions, cumbersome marking process
^68^Ga	PET	67.71 min	1.920 MeV	^68^Zn(p,n)^68^Ga	preclinical/clinical	convenient and easy to prepare, currently the most commonly used radionuclide for GLP-1R imaging
^89^Zr	PET	78.4 h	395.5 KeV	^89^Y(p,n)^89^Zr	preclinical	Limited stability due to covalent bond breakage between chelator and protein
^177^Lu	SPECT	6.7 days	β-0.497 MeV γ 0.208 MeV	^176^Lu(n,γ)^177^Lu	preclinical	labeled GLP1R, whose substantial uptake in the kidney limits its potential for further conversion

The current treatment modality is still mainly surgical excision. Therefore finding an integrated approach to GLP1-R molecular medicine treatment as soon as possible could provide more guidance to clinicians in their choice of intervention strategies. Can we explore the feasibility of GLP-1R molecular imaging in other diseases or fields and open the gateway to push on it? With this in mind, we have summarised and reviewed the current status of GLP-1R molecular imaging in other fields, and it may be an opportunity to explore its theranostic.

### 3.1 Other neoplasms

SSTR is highly expressed in malignant tumors but rarely in GLP-1R. The results of Bongetti’s research (Bongetti et al., 2018) gave us a great warning. Although benign insulinoma accounts for 90% of all insulinomas, 10% are still malignant. Therefore, for patients with negative imaging results who remain highly suspected of insulinoma, we should be alert to whether they are malignant. At this time, we need to take the receptor imaging technology of SSTR to identify them, such as 68Ga-Dotatate-exendin-4. Do not miss or misdiagnose the patients with negative GLP-1R imaging. The study of Korner et al. (2007), other than insulinoma, illustrated that GLP-1R is also high expression in gastrinoma, MTC, 60% of pheochromocytoma, and gastrinoma. Given this, is molecular imaging targeting GLP-1 analogs feasible in these tumors? A study has since given partial answers.

Pach et al. (2013) performed [Lys (Sood et al., 2019) (Ahx-HYNIC-(99m)Tc/EDDA)NH_2_]-Exendin-4 SPECT/CT imaging in four enrolled MTC patients for the first time. These results displayed that GLP-1R molecular imaging is not exclusive to insulinoma and plays a vital role in other diseases. Clinical experience has pointed to these tumors as high in receptor expression, and they all have a relatively low clinical incidence. Even with pheochromocytoma, which has a relatively high prevalence, the more established SSTR molecular imaging agent DOTATATE is now available. Hence, these factors have directly or indirectly hindered the exploration of GLP-1R molecular imaging in tumor diseases other than insulinoma. It is both a challenge and an opportunity, so searching for additional molecular probes may present promising results in these oncological diseases.

### 3.2 Myocardial ischemia

Currently, the most commonly used methods for the assessment of surviving myocardium after myocardial infarction in clinical practice include EHCO, CAG, CTCA, and MRI. In the past, these methods were used to assess myocardial survival after infarction from the level of myocardial dynamics and perfusion, but they were unable to analyze the metabolic level of the surviving myocardium from the molecular metabolism point of view in a more in-depth manner. The FDG currently used in PET/CT is taken up by myocardium with metabolic activity but not by necrotic myocardium. Thus, myocardial metabolism can be evaluated by this mechanism. Also PET dynamic myocardial perfusion imaging agents combined with 13N-NH3 provide more accurate judgment (Kim et al., 2015). In the cardiovascular, GLP-1R can improve cardiovascular function and reduce inflammation (Laviola et al., 2012). Moreover, it is involved in the protective effect of myocardial cells after myocardial ischemia. So, can infarcted myocardial cells be targeted by nuclide-labeled GLP-1R molecular imaging? The publication of two critical studies has also delivered on the promise of GLP-1R imaging in cardiovascular disease.

In the myocardial ischemia/reperfusion (MI/R) study (Gao et al., 2012), 18F-FBEM-Cys40-exendin-4 could monitor the dynamic changes of GLP-1R upregulation in the myocardial infarction/ischemia area. Meanwhile, the uptake changes observed by PET were consistent with the changes in receptor expression by Western blot analysis and histochemistry. Its protective mechanism and analogs in myocardial cells with ischemia injury are now better-understood thanks to this report, which is the first to image GLP-1R in myocardial cells that have undergone MI/R. It marks the triumphant entry of molecular imaging into the cardiovascular field. Another blockbuster study (Stahle et al., 2020) was the first application of the 68Ga-NODAGA-exendin-4 molecular probe in a myocardial infarction model. The encouraging conclusion indicated that the uptake of 68Ga-NODAGA-exendin-4 in the infarcted area was increased by 8.6 times compared with the sham operation area. The uptake of the imaging agent was correlated with the number of CD68-positive macrophages in the infarcted area and α-smooth muscle actin staining in the distal myocardium. Molecular imaging can dynamically monitor the protective effect of GLP-1R on ischemic cardiomyocytes, which brings a new auxiliary method for evaluating the therapeutic effect of myocardial ischemia and myocardial infarction.

And compared with FDG PET/CT, GLP1R molecular imaging is more friendly to patients with type 2 diabetes. It allows for a more visual assessment of GLP1R metabolic expression levels in the surviving myocardium at the molecular metabolic level. Therefore, in the future, it could complement the assessment of inflammation in the molecular metabolism of surviving post-infarction cardiomyocytes. And it can be used together with other routine tests to provide the evaluation value of surviving post-infarction cardiomyocytes from different perspectives including myocardial dynamics, perfusion and molecular metabolism. However, because only a few preclinical studies of GLP1R molecular imaging have been performed, there is a need for a larger future.

### 3.3 Endocrine-related fields

GLP-1/GLP-1R in the endocrine field has been accompanied by the introduction of semaglutide, which has become a principal receptor target. The initial release of the SUSTAIN and PIONEER phase III clinical trials established its landmark importance in treating type 2 diabetes mellitus (T2DM) and obesity (Meier, 2021). More recently, research on this new GLP-1 analog has gradually expanded into other areas, including diabetes, chronic kidney disease, cardiovascular disease, NASH, and Alzheimer’s disease (Knudsen and Lau, 2019). However, no molecular imaging studies with semaglutide have been performed, and the endocrine field is still dependent on exendin-4 and its derivatives for molecular imaging. This section reviews the progress and emerging opportunities for endocrine-related GLP-1R molecular imaging.

#### 3.3.1 Quantified pancreatic beta (β)-cell mass (BCM) in diabetes

It is well known that a decrease in pancreatic islet beta cells causes diabetes (Moonschi et al., 2017). At first, unlabeled GLP-1 analogs were developed for glycemic control in patients with diabetes, whereas pancreatic beta-cell mass (BCM) is essential for controlling and treating diabetes. Visual evaluation of BCM was invasive in the past (Murakami et al., 2021b). Non-invasive molecular imaging of radionuclides targeting GLP-1R in nuclear medicine has brought light to the quantitative evaluation of BCM. It directly drives progress in endocrinological diabetes research (Table 4).

**TABLE 4:** Summary of GLP1R molecular imaging in Endocrine diseases.

Year	Chelator	Compound	Preclinical/Clinical	Object	Significance	Deficiency	References
2015, 2016	NOTA	68Ga-NOTA-exendin-4	clinical	MEN-1	successfully identified multiple small insulinomas in confirmed MEN-1 patients	Case studies lack large sample data support	Luo et al. (2015), Luo et al. (2016a)
2012	FBA	[18F]Ex (9–39)	preclinical	quantified diabetes pancreatic BCM	first successful quantification of BCM in SD rats and BioBreeding diabetes-prone (BB-DP) rats	None of the three studies used exendin-4 as a molecular probe for comparison	Wang et al. (2012)
2018	FBA	[18F]FB40-Ex (9–39)	preclinical	quantified diabetes pancreatic BCM	Developed derivatives for PET imaging of pancreatic beta cells		Kimura et al. (2018)
2012	DOTA	[64Cu](Lys40 (DOTA)NH2)-exendin-4	preclinical	quantified diabetes pancreatic BCM	64Cu labeled GLP1R imaging was first used in BCM	high uptake in the kidney, which seriously affected the quantitative determination of BCM	Connolly et al. (2012)
2014, 2017	DTPA	([Lys12(111In-BnDTPA-Ahx)]exendin-4)	preclinical	quantified diabetes pancreatic BCM	111In labeled GLP1R imaging was successfully applied in BCM and guided the treatment of diabetic mice	No further clinical translational studies	Brom et al. (2014), Kimura et al. (2017)
2017	DO3A	[177Lu]Lu-DO3A-VS-Cys40Exendin-4	preclinical	pancreatic uptake in various species	the crucial concept of IPR was proposed. IPR has also become an influential statistical factor in imaging pancreatic ginning to make their mBCM	Physiological uptake in different species has not been compared, and related diseases have not been studied	Eriksson et al. (2017)
2017	None	111In-Exendin-3	clinical	heterotopic islet transplantation	111in-Exendin-3 imaging to detect the quality of transplanted BCM and measure the volume using C3H of heterotopic islet transplantation in the calf muscle of mice	Exendin-4 control studies not performed, small sample case reports	Eter et al. (2017)
2016	DTPA	[Lys40 (Ahx-DTPA-111In) NH2]	clinical	left brachioradialis islet transplantation	Different nuclide labeling exendin-4 GLP1R imaging completed BCM measurement in islet transplantation	Case reports with only a small sample size are not convincing	Wu et al. (2013), Li et al. (2020), Espes et al. (2016), Pattou et al. (2010)
2013	TTCO	[18F]F-TTCO-Cys (40)-exendin-4	clinical	graft in liver of islet transplantation			
2010	DO3A	[177Lu]DO3A-VS-Cys40-Exendin-4	preclinical	islet transplantation mice model			
2020	PTTCO	[18F]F-PTTCO-Cys 40-Exendin-4	clinical	portal vein grafted islets			
2021	None	MnMEIO NPs	preclinical	Min-6 β-cells in the recipients after transplantation	study on that combination of exendin-4 and islet transplantation for the first time	Unlabeled radionuclides for imaging researches	Juang et al. (2021)
2022	NODAGA	68Ga-NODAGA-exendin-4	clinical	congenital hyperinsulinemia (CHI)	the first to discuss that 68Ga-NODAGA-exendin-4 PET/CT has a higher sensitivity than 18F-DOPA-exendin-4 for the preoperative localization of CHI lesions	Lack of comparative studies between 68Ga chelators such as DOTA, NODAGA, DO3A and NOTA	Boss et al. (2022), C et al. (2020)
2020	DOPA	18F-DOPA-exendin-4	clinical	congenital hyperinsulinemia (CHI)			
2020	DOTA	68Ga-DOTA-exendin-4	clinical	congenital hyperinsulinemia (CHI)	First used 68GaDOTA-exendin-4 PET/CT to visualize CHI lesions successfully		
2021	DOTA	68Ga-DOTA-Exendin-4	clinical	congenital hyperinsulinemia (CHI)	Successful detection of 2 of 3 CHI in 20 EHH patients via 68Ga-DOTA-exendin-4	Lack of controlled and theranostic studies	Kalff et al. (2021)
2019	DOTA	68Ga-DOTA-exendin-4	clinical	MEN-1	GLP1R imaging combined with MRI is a feasible method to detect the lesions of MEN-1 patients and guide the surgical treatment	No comparison of PET/CT with SPECT/CT was performed, nor between different chelators labeled with 68Ga	Antwi et al. (2019)
2019	Dotatate	68Ga-Dotatate-exendin-4	clinical	insulinoma and MEN-1	SSTR imaging has higer sensitivity in malignant insulinoma patients than MEN-1	SSTR expression was not quantified in MEN-1 patients	Shah et al. (2022)
2020	NODAGA	68Ga-NODAGA-exendin-4	clinical	Obesity	Detecting the difference in GLP1R intracranial distribution of obese patients compared with ordinary people	This tracer was not suitable for GLP1R distribution imaging in the brain of these patients	Deden et al. (2021)

The GLP-1R antagonist exendin (Gotthardt et al., 2002; Wild et al., 2006; Wicki et al., 2007; Vegt et al., 2008; Wild et al., 2008; Christ et al., 2009; Brom et al., 2010; Wild et al., 2010; Wild et al., 2011; Wu et al., 2011; Kiesewetter et al., 2012a; Kiesewetter et al., 2012b; Keliher et al., 2012; Christ et al., 2013; Sowa-Staszczak et al., 2013; Wu et al., 2013; Yue et al., 2013; Sel et al., 2014; Wu et al., 2014; Antwi et al., 2015; Bauman et al., 2015; Xu et al., 2015; Falconi et al., 2016; Sowa-Staszczak et al., 2016; Kaeppeli et al., 2019; Zhang et al., 2019; Li et al., 2020; Mori et al., 2020; Senica et al., 2020; Murakami et al., 2021a; Qiu et al., 2023) is the most widely applied in diabetic BCM. It is a truncated form of the GLP-1 agonist exendin-4 and can significantly reduce the insulin-to-glucose ratio without affecting glucose tolerance or insulin sensitivity (Blevins et al., 2014). Earliest, Wang (Wang et al., 2012) combined 18F and FBA to 6-hydroxynitrile aldehyde at Lys27 of Exendin (Gotthardt et al., 2002; Wild et al., 2006; Wicki et al., 2007; Vegt et al., 2008; Wild et al., 2008; Christ et al., 2009; Brom et al., 2010; Wild et al., 2010; Wild et al., 2011; Wu et al., 2011; Kiesewetter et al., 2012a; Kiesewetter et al., 2012b; Keliher et al., 2012; Christ et al., 2013; Sowa-Staszczak et al., 2013; Wu et al., 2013; Yue et al., 2013; Sel et al., 2014; Wu et al., 2014; Antwi et al., 2015; Bauman et al., 2015; Xu et al., 2015; Falconi et al., 2016; Sowa-Staszczak et al., 2016; Kaeppeli et al., 2019; Zhang et al., 2019; Li et al., 2020; Mori et al., 2020; Senica et al., 2020; Murakami et al., 2021a; Qiu et al., 2023), synthesized the first molecular developer [18F]Ex (Gotthardt et al., 2002; Wild et al., 2006; Wicki et al., 2007; Vegt et al., 2008; Wild et al., 2008; Christ et al., 2009; Brom et al., 2010; Wild et al., 2010; Wild et al., 2011; Wu et al., 2011; Kiesewetter et al., 2012a; Kiesewetter et al., 2012b; Keliher et al., 2012; Christ et al., 2013; Sowa-Staszczak et al., 2013; Wu et al., 2013; Yue et al., 2013; Sel et al., 2014; Wu et al., 2014; Antwi et al., 2015; Bauman et al., 2015; Xu et al., 2015; Falconi et al., 2016; Sowa-Staszczak et al., 2016; Kaeppeli et al., 2019; Zhang et al., 2019; Li et al., 2020; Mori et al., 2020; Senica et al., 2020; Murakami et al., 2021a; Qiu et al., 2023) for quantifying BCM in the bio breeding diabetes-prone (BB-DP) animal model. The following year, Kimura (Kimura et al., 2018) exploited [18F]-FB40-Ex (Gotthardt et al., 2002; Wild et al., 2006; Wicki et al., 2007; Vegt et al., 2008; Wild et al., 2008; Christ et al., 2009; Brom et al., 2010; Wild et al., 2010; Wild et al., 2011; Wu et al., 2011; Kiesewetter et al., 2012a; Kiesewetter et al., 2012b; Keliher et al., 2012; Christ et al., 2013; Sowa-Staszczak et al., 2013; Wu et al., 2013; Yue et al., 2013; Sel et al., 2014; Wu et al., 2014; Antwi et al., 2015; Bauman et al., 2015; Xu et al., 2015; Falconi et al., 2016; Sowa-Staszczak et al., 2016; Kaeppeli et al., 2019; Zhang et al., 2019; Li et al., 2020; Mori et al., 2020; Senica et al., 2020; Murakami et al., 2021a; Qiu et al., 2023) for the quantification of diabetic BCM. It had a stronger targeting affinity and could visualize pancreatic β-cells. The initial conditions for a molecular probe for GLP-1 R imaging were fulfilled, and a new frontier was opened for non-invasive molecular imaging. Continuing the heat of exendin (Gotthardt et al., 2002; Wild et al., 2006; Wicki et al., 2007; Vegt et al., 2008; Wild et al., 2008; Christ et al., 2009; Brom et al., 2010; Wild et al., 2010; Wild et al., 2011; Wu et al., 2011; Kiesewetter et al., 2012a; Kiesewetter et al., 2012b; Keliher et al., 2012; Christ et al., 2013; Sowa-Staszczak et al., 2013; Wu et al., 2013; Yue et al., 2013; Sel et al., 2014; Wu et al., 2014; Antwi et al., 2015; Bauman et al., 2015; Xu et al., 2015; Falconi et al., 2016; Sowa-Staszczak et al., 2016; Kaeppeli et al., 2019; Zhang et al., 2019; Li et al., 2020; Mori et al., 2020; Senica et al., 2020; Murakami et al., 2021a; Qiu et al., 2023) quantifying the diabetic BCM, molecular imaging trials of exendin-4 in this field are also in full swing. Connolly (Connolly et al., 2012) is the first to attempt the composite 64Cu-DOTA-exendin-4. However, it was impacted by the tracer’s high uptake in the kidney, which obscured part of the pancreatic tissue and seriously affected the quantitative determination of BCM. Until two diabetes model studies (Brom et al., 2014; Kimura et al., 2017) completed the quantification of BCM under the derivatives ([Lys12(111In-BnDTPA-Ahx)]exendin-4) and guided the treatment of diabetes. It also signifies the value and significance of GLP-1R molecular imaging.

177-Lu, as an essential part of theranostics, does not perform satisfactorily in previous insulinoma imaging. Eriksson (Eriksson et al., 2017) initially applied [177Lu]Lu-DO3A-VS-Cys40Exendin-4 to the comparative study of pancreatic uptake in various species. The ratio of islets to exocrine pancreas varies between species; consequently, the crucial concept of IPR was proposed. IPR has also become an influential statistical factor in imaging pancreatic β-cells and determining BCM. Such results are promising. Compared to the therapeutic limitations of insulinoma, quantifying BCM and treating diabetes through GLP-1R molecular imaging have shed light on theranostics.

#### 3.3.2 Islet transplantation

Before the development of mesenchymal stem cells and pluripotent stem cells for the treatment of diabetes, the most effective treatment for type 1 diabetes (T1D) was portal vein islet transplantation. It primarily administered donor islets to the liver via the portal vein. In the hepatic sinusoids, the islet cells could proliferate and secrete insulin. Portal vein islet transplantation has achieved good results in protecting patients from severe hypoglycemia. Regarding pancreatic β-cells, it makes sense to monitor graft survival in patients with pancreatic β-cell transplantation with GLP-1R molecular imaging. Previous studies have clarified (Hubalewska-Dydejczyk et al., 2015) that GLP-1R imaging helps monitor pancreatic beta cell quality in patients with fragile type I diabetes after islet transplantation. Indeed, few articles exist on molecular imaging of transplanted islets.

Initially, 111In-Exendin-3 SPECT/CT imaging (Eter et al., 2017) detected the quality of transplanted β-cells and measured the volume using C3H of heterotopic islet transplantation in the calf muscle. Pattou et al. (2010) featured a left brachioradialis islet transplantation case, and GLP-1R molecular imaging with Lys40 (Ahx-DTPA-111In)NH2-exendin-4 revealed locally high uptake in the left forearm islet graft. According to these two reports, 111In-exendin SPECT/CT imaging is anticipated to become a novel molecular probe for non-invasive quantitative detection of β-cell in human islet transplantation. In addition, several other radionuclide markers for islet graft imaging have been developed. Such as (Yue et al., 2013) F-TTCO-Cys (Sood et al., 2019)-exendin-4 (Wu et al., 2013) visualize the graft in liver imaging of islet transplantation, similar results were obtained for 177Lu-DO3A-Exendin-4 (Espes et al., 2016) and 18F-PTTCO-Exendin-4 (Li et al., 2020). These studies lack data from sufficiently large sample sizes, and the case studies remain inconclusive.

Moreover, a recent study (Juang et al., 2021) showed the interaction between manganese with engineered iron oxide nanoparticles (MnMEIO NPs) and Exendin-4. Successfully detecting the labeled Min-6 β-cells in the recipients after transplantation in combination with MRI. Unfortunately, they did not target labeling radionuclides. Exploring more GLP-1R derivatives and other analogs for islet transplantation has become necessary. Monitoring transplanted pancreatic β-cells to guide graft survival is a new benefit for islet transplantation patients. Such a gap also represents a worthwhile direction for future research in GLP-1R molecular imaging.

#### 3.3.3 Congenital hyperinsulinemia (CHI)

Benign insulinoma is the most common cause of endogenous hyperinsulinemic hypoglycemia (EHH). GLP-1R molecular imaging will become the ideal choice for non-invasive localization and diagnosis of benign insulinoma patients in the future. The clinical presentation of CHI is similar to that of insulinoma, which can cause misdiagnosis. It must therefore be carefully excluded when diagnosing insulinoma with EHH symptoms. In the past, nesidioblastosis was synonymous with congenital hyperinsulinemia. Until 1995 (Ma et al., 2020), the genetic basis of CHI was thought to be an inactivating mutation in the subunit. That forms the β-cell plasma membrane ATP-dependent potassium channel. Thus, it is not the proliferation or the hyperinsulinemia of β-cell proliferation. Hence the name is defined as CHI. It is more common in neonates and children, but Shah et al. (2017) reported that it could also appear in adults. Due to the different treatment options between benign insulinoma and CHI, accurately identifying the two diseases can avoid omission and misdiagnosis to a greater extent.

Boss et al. (2022) compared the sensitivity of 68Ga-NODAGA-exendin-4 and 18F-DOPA-exendin-4 PET/CT for the diagnostic efficacy of 19 patients with CHI. The sensitivity of 68Ga-NODAGA-exendin-4 at 100% was significantly higher than that of 18F-DOPA-exendin-4 at 71%. It is the first to discuss whether GLP-1R molecular imaging is a good choice for the preoperative localization of CHI lesions. The research that made a molecular imaging breakthrough in this field is credited to Christ (C et al., 2020). In the study, the pancreatic islet cells with CHI lesions had an extreme expression of GLP-1R. He discovered that the intensity of CHI uptake in PET molecular images ranged between insulinoma and normal pancreatic tissue. It provides a more convenient non-invasive test for differentiating benign insulinoma and CHI. This result has brought a historic breakthrough in GLP-1R molecular imaging.

In recent years, several studies (Reubi et al., 2010; Kalff et al., 2021) using different molecular probes to visualize CHI presented results similar to Christ’s. Interestingly, a trial with a negative outcome caught our attention (Reubi et al., 2010). The results indicated that patients with the non-insulinoma pancreatic hypoglycemic syndrome (NIPHS) and post-gastric bypass hypoglycemic syndrome (PGBH) could not be distinguished via GLP-1R molecular imaging. A plausible explanation may be that in these diseases, EHH symptoms prevent pancreatic cells from over-expressing this receptor. Its molecular imaging offers a new diagnostic option for insulinomas and CHI, which are rare and hard to identify in clinical practice. Nevertheless, molecular imaging still has limitations and does not apply to patients with specific etiologies of EHH, such as NIPHS and PGBH. The road ahead for molecular medicine remains daunting, and we need to focus on trial and error in these gap areas.

#### 3.3.4 Multiple endocrine neoplasia type 1 (MEN-1)

MEN-1 is an autosomal dominant genetic disease caused by mutations in its tumor suppressor gene. Most patients will gradually progress to multifocal functional or non-functional pancreatic neuroendocrine tumors. Therefore, seeking a non-invasive means of identification at an early stage is necessary. GLP-1R molecular imaging in patients with MEN-1 mutations is a subject of increasing research. It is a very positive sign for endocrinology. Molecular imaging can enhance the diagnosis of an ion channel or genetically inherited diseases such as CHI and MEN-1, which are rare in clinical practice.

In 2019, Antwi et al. (2019) studied six MEN-1 patients diagnosed by genetic testing among 52 recruited EHH patients. The sensitivity of 68Ga-DOTA-exendin-4 PET/CT combined with MRI (92.3%) is significantly higher than MRI (38.5%), and PET/CT (84.6%) alone, GLP-1R molecular imaging combined with MRI is a feasible method to detect the lesions of MEN-1 patients and guide the surgical treatment. Subsequently, two cases published (Luo et al., 2015; Luo et al., 2016a) successfully identified multiple insulinomas in confirmed MEN-1 patients with the tracer 68Ga-NOTA-exendin-4 PET/CT imaging. GLP-1R molecular imaging has become a potential direction for diagnosing insulinoma in MEN-1.

That said, the results of an earlier study (Shah et al., 2022) are worth considering. SSTR-targeted tracer 68Ga-DOTATATE PET/CT was utilized to compare the lesions in insulinomas and MEN-1 patients. The tracer had significantly higher sensitivity and positive predictive value in malignant insulinoma. It could guide the theranostic PRRT treatment other than MEN-1. SSTR molecular imaging is not applicable in MEN-1, so the value of GLP-1R molecular imaging for this disease becomes even more valuable. Furthermore, a first meta-analysis (Shah et al., 2021) last year concluded that GLP-1R could act as a molecular probe for accurate preoperative localization imaging in MEN-1 patients after summarizing all previous cases of MEN-1 patients. Due to the difficulty and rarity of diagnosing MEN-1 patients, the predictive value of GLP-1R imaging prior to pathological examination is especially relevant. Nonetheless, the labeled nuclide in MEN-1 continues to be 68Ga. Research on long half-life therapeutic nuclides such as 177Lu is still lacking, and it is challenging to integrate molecular medicine theranostics in this field.

In summary, from our summary of the current status of GLP-1R molecular imaging in endocrinology (Table 4), we realize that quantification of BCM in diabetes and islet transplantation may be the last winner and that current advances in molecular medicine in this field are full of promise. Diseases such as CHI and MEN-1, which are clinically uncommon and easily underdiagnosed, have also been studied and molecular imaging has given them crossover implications. We therefore have reason to believe that non-invasive molecular medicine in endocrinology may lead to a remarkable achievement in the theranostic.

### 3.4 Neurodegenerative disease

The expression of GLP-1R was initially detected in the nucleus tractus solitarius and hypothalamus. As the research progressed, so did other brain parts, such as the frontal cortex, hippocampus, amygdala, and substantia nigra. In addition to being primarily distributed in the pancreatic islet cell, it can appear selectively in the central nervous system (Jensen et al., 2018; Mansur et al., 2019; Gabery et al., 2020). The theranostic of central nervous system diseases targeting GLP-1R has been gradually developed. It is also a key area of focus for molecular medicine at this stage.

Molecular imaging has already become a crucial clinical tool in the diagnosis of AD and PD. For instance, the tracer 11C-PIB, as is now widely used for imaging AD age spots (Klunk et al., 2004). However, 11C has a half-life of only 20 min, limiting its use in routine examinations. 18F has a longer half-life. Therefore 18F-based PET imaging began a long quest in the disease assessment of AD. From the initial low specificity of FDG PET for pathological changes in AD to the drawbacks that amyloid PET imaging does not correlate with the severity of the disease. The unfolding of the Tau PET examination has provided significant value in the assessment of early pathological changes and disease severity in AD. And later with the 18F-florbetapir have been employed in AD as beta-amyloid (Aβ) imaging agents (Clark et al., 2012), while 18F-FDOPA has been used in PD diagnosis (Brooks, 2010). Two previous studies in PD models (Bertilsson et al., 2008; Isacson et al., 2011) have confirmed that exendin-4 can restore the nerve in the subventricular zone and hippocampus, thereby improving mice’s cognitive, motor, and memory abilities. Bu et al. (2021) still bound exendin-4 with AAV-9-A53T-α-synuclein in the PD model and observed that exendin-4 treatment attenuated the pathological accumulation of α-synuclein. In recent years, additional studies (Salameh et al., 2020; Lv et al., 2021; Zhang et al., 2022) have found that incretin receptor agonists (Exendin-4 and DA-JC4) and GLP-1/GIP dual receptor agonists DA5-CH can successfully cross the blood-brain barrier and reach the pathological area of PD, which is expected to become an ideal therapeutic drug. Exendin-4 has shown excellent results in improving cognitive function in Parkinson’s disease. The mechanism and efficacy of a novel GLP-1R analog liraglutide in PD are also evaluated (Kim et al., 2017; Mah et al., 2022; Chen et al., 2023). GLP-1R targets may usher in a new chapter in the theranostic of central nervous system diseases.

**FIGURE 6:**
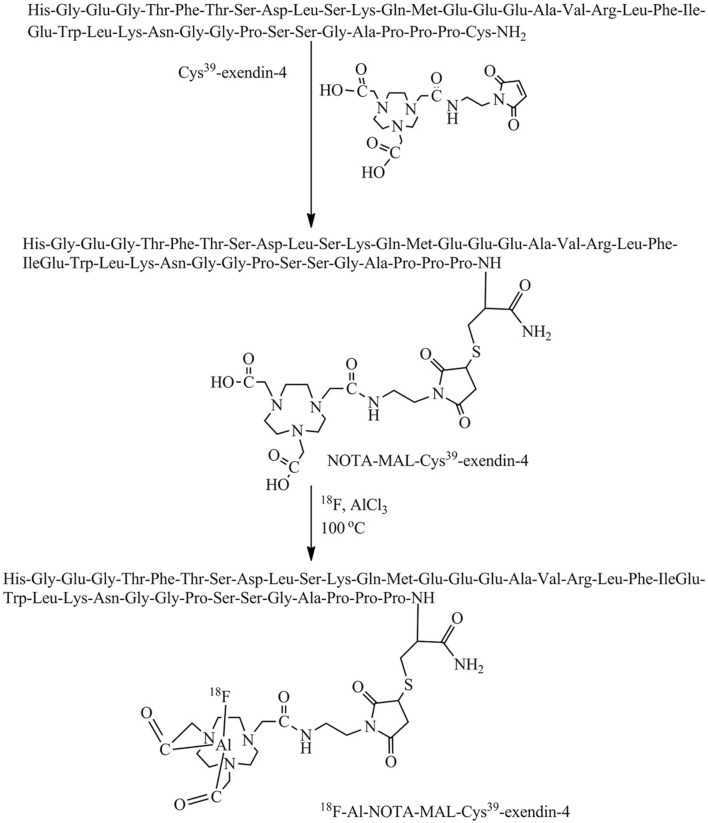
Scheme for the synthesis of [18F]AlF-NOTA-MAL-Cys39-exendin-4. Modified by coupling Cys39-exendin-4 to NOTA-MAL by addition of ionized target water containing 18F (740 MBq). Heated at 100°C for 10 min. Cool and dilute the mixture with sterile water and inject into a validated Varian BondElut C18 cartridge. 10 mL of sterile water washes the column twice and ethanol hydrochloride (0.3 mL) elutes the labelled peptide.

For the first time, Wang et al. (2018) illustrated the effect of age differences on GLP-1R distribution in the brain by synthesize [18F]AlF-NOTA-MAL-Cys39-exendin-4, the peptide was modified by coupling Cys39-exendin-4 to NOTA-MAL and was successfully synthesized using 18F-targeted labeling of the peptide(Figure 6). Its expression in the brains of aged mice was elevated reduced. The discovery of this molecular probe creates a better prospect for applying GLP-1R molecular imaging to neurodegenerative diseases such as PD and AD. In 2020, Liu et al. (2020) published the investigation via PET imaging in Alzheimer’s disease (AD) model, different derivatives with 18F labeling followed by microPET. [18F]FBEM-exendin-4 is more likely to cross the blood-brain barrier due to its better lipid solubility than [18F]AIF-exendin-4 based on previous findings. [18F]FBEM-Cys39-exendin-4 was compared with three other 18F-labeled derivatives of PET imaging. This tracer successfully evaluated the decrease of total GLP-1R expression in the AD model. It is the first report of GLP-1R molecular imaging in patients with neurodegenerative disease, which opens a good direction for the future in this still-unknown field. Deden et al. (2021) also used a 68Ga-labeled tracer to detect the difference in receptor intracranial distribution of obese patients compared with ordinary people. Unfortunately, 68Ga-NODAGA-exendin-4 PET was unsuitable for GLP-1R distribution imaging in the brain of obese patients.

The research on GLP-1R molecular imaging in neurodegenerative diseases and other central nervous system diseases has not achieved more extraordinary breakthroughs, limited to preclinical studies and related studies of blood-brain barrier penetration. There remains a significant gap in the comparative study of GLP-1R and neurodegenerative mechanisms and the improvement of symptoms in clinical patients. The experimental investigation of combining GLP-1R molecular imaging with other related nervous system diseases is an emerging opportunity in that uncharted territory. It could drive the neurology theranostic.

## 4 GLP1R molecular visualization of ligands and chelator

As mentioned previously, ligand selection for GLP-1R molecular imaging is currently still centred on exendin-4. Although exendin-3 and exendin-(9–39) have also been involved as other ligands in studies of insulinoma and pancreatic β-cell mass determination. However, the structural form of the ligand for both exists only in the single form of Lys40-exendin-3, Lys27-exendin-(9–39) (Gasbjerg et al., 2021). Diverse ligand forms are currently being designed and developed around exendin-4. These include Lys39-exendin-4, Lys40-exendin-4, Nle14, Lys40-exenidn-4, Cys40-exendin-4 and other different ligand types. Table 5 lists the different types of ligands designed for GLP-1R molecular imaging and their corresponding radiolabels, linkers, conjugation structures, etc. Lys40-exendin-4 and Cys40-exendin-4 are the two most established and widely used ligands. They can show good stability with different chelating agents. In terms of linkers, Ahx has almost become the obvious choice for GLP-1R molecular imaging. The introduction of the hydrophobic linker Ahx between the peptide and the marker effectively protects the stability of the labelled peptide. It makes the conjugated structure and the peptide more stable, which facilitates the progress of subsequent studies.

**TABLE 5:** List of molecular probes targeting GLP1R.

Ligand	Chelator	Linker	Radionuclide	Conjugate	Compound (abbreviation)	Application
Lys^40^-exendin-3	DOTA	Ahx	^68^Ga	[Lys^40^(Ahx-DOTA)NH_2_]-exendin-3	^68^Ga-DOTA-exendin-3	preclinical/clinical
Lys^12^-exendin-4	BnDTPA	Ahx	^111^In	[Lys^12^(BnDTPA-Ahx)]-exendin-4	^111^In-BnDTPA-exendin-4	preclinical
Lys^40^-exendin-4	DTPA	Ahx	^111^In	[Lys^40^(Ahx-DTPA)NH_2_]-exendin-4	^111^In-DTPA-exendin-4	preclinical/clinical
Lys^40^-exendin-4	DOTA	Ahx	^111^In, ^68^Ga, ^64^Cu	[Lys^40^(Ahx-DOTA)NH_2_]-exendin-4	^111^In-DOTA-exendin-4 ^68^Ga-DOTA-exendin-4 ^64^Cu-DOTA-exendin-4	preclinical/clinical(^111^In, ^68^Ga) preclinical (^64^Cu)
Lys^40^-exendin-4	HYNIC/EDDA	Ahx	^99m^Tc	[Lys^40^(Ahx-HYNIC/EDDA)NH_2_]-exendin-4	^99^mTc-HYNIC-exendin-4	preclinical/clinical
Lys^40^-exendin-4	DFO	Ahx	^89^Zr	[Lys^40^(Ahx-DFO)NH_2_]-exendin-4	^89^Zr-DFO-exendin-4	preclinical
Nle^14^, Lys^40^-exendin-4	DOTA	Ahx	^111^In, ^68^Ga	[Nle^14^-Lys^40^-(Ahx-DOTA)NH_2_]-exendin-4	^111^In-DOTA-exendin-4 ^68^Ga-DOTA-exendin-4	preclinical/clinical
Nle^14^, Lys^40^-exendin-4	NODAGA	—	^68^Ga	[Nle^14^-Lys^40^-(NODAGA)NH_2_]-exendin-4	^68^Ga-NODAGA-exendin-4	preclinical/clinical
Cys^39^-exendin-4	NOTA	MAL	^18^F, ^68^Ga	[Cys^39^(MAL-NOTA)NH_2_]-exendin-4	^18^F-NOTA-exendin-4 ^68^Ga-NOTA-exendin-4	preclinical case reports (^68^Ga)
Lys^27^-exendin-(9–39)	FBA	—	^18^F	[Lys^27^-(FBA)NH_2_]-exendin-(9–39)	[^18^F]Ex (9–39) [^18^F]FB40-Ex (9–39)	preclinical
Cys^40^-exendin-4	DO3A	VS	^68^Ga, ^64^Cu	[Cys^40^-(VS-DO3A)OH]-exendin-4	^68^Ga-DO3A-VS-Cys40-exendin-4 ^64^Cu-DO3A-VS-Cys40-exendin-4	preclinical/clinical (^68^Ga) preclinical (^64^Cu)
Cys^40^-exendin-4	BaMalSar	—	^64^Cu	[Cys^40^-(BaMalSar)NH_2_]-exendin-4	^64^Cu-BaMalSar-exendin-4	preclinical
Cys^40^-exendin-4	Mal_2_Sar	—	^64^Cu	[Cys^40^-(Mal_2_Sar)NH_2_]-exendin-4	^64^Cu-Mal_2_Sar-exendin-4	preclinical
Cys^40^-exendin-4	DO3A	VS	^177^Lu	[Cys^40^-(VS-DO3A)OH]-exendin-4	^177^Lu-DO3A-VS-Cys40-exendin-4	preclinical
Cys^40^-exendin-4	NOTA	MAL	^18^F	[Cys^40^-(MAL-NOTA)NH_2_]-exendin-4	^18^F-AlF-NOTA-MAL-cys40-exendin-4	preclinical
Cys^40^-exendin-4	FBEM	—	^18^F	[Cys^40^-(FBEM)NH_2_]-exendin-4	^18^F-FBEM-cys40-exendin-4	preclinical
Cys^40^-exendin-4	TCO/TTCO	—	^18^F	[Cys^40^-(TCO/TTCO)NH_2_]-exendin-4	^18^F-TCO/TTCO-cys40-exendin-4	preclinical
Cys^40^-exendin-4	PEG	—	^18^F	[Cys^40^-(PEG)NH_2_]-exendin-4	^18^F-FEG-cys40-exendin-4	preclinical

**FIGURE 7:**
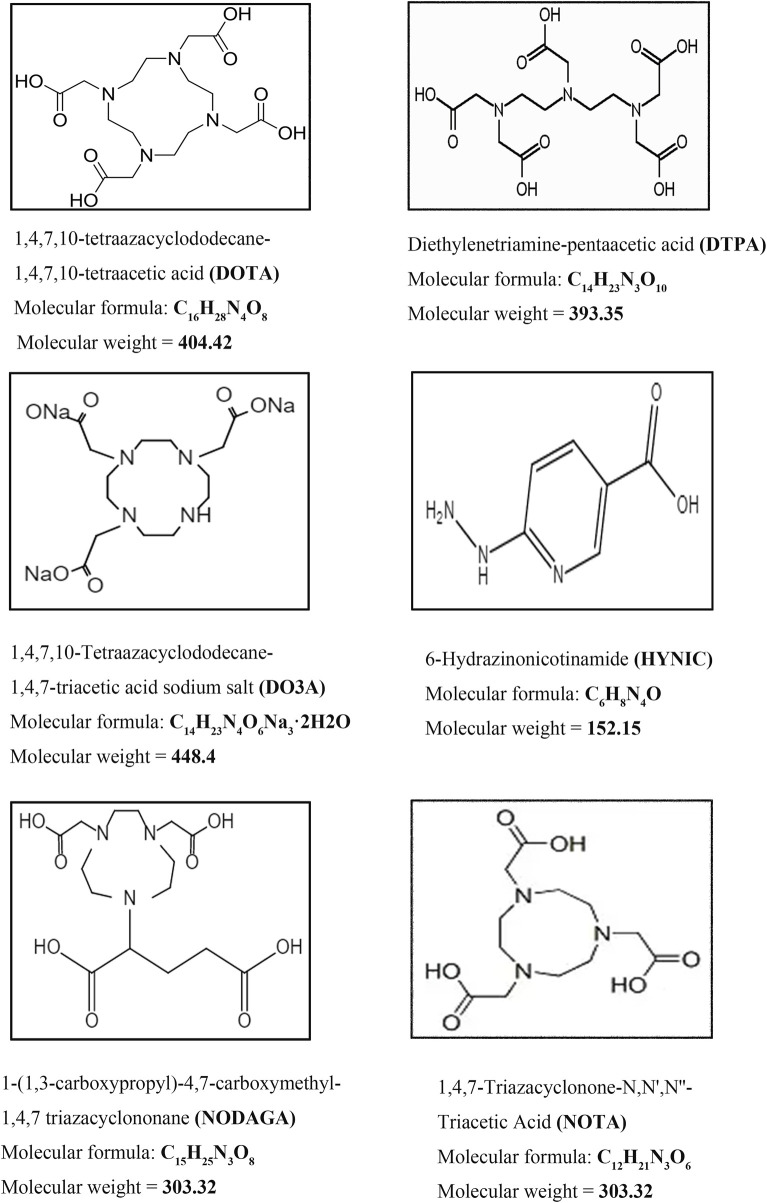
Commonly used chelating agents in GLP-1R molecular probes. The most classic chelators, DOTA, DTPA and the ^99m^Tc HYNIC chelator, which were used in the first GLP-1R molecular imaging studies, have all been successfully translated and used in clinical studies. On this basis, the advantages of ^68^Ga radionuclides in GLP-1R imaging have been highlighted. New chelators, including DO3A, NOTA and NODAGA, are also beginning to make their mark in GLP-1R molecular imaging.

A chelator is an organic molecule that can chelate with heavy metal ions. Both SPECT/CT and PET/CT present a more diverse selection of chelating agents to try in preclinical studies. Unfortunately the chelating agents that eventually translate to clinical use are mainly shown in Figure 7. One of the smallest molecular weights, Hydrazinonicontinamide (HYNIC), is an established bifunctional complexing agent. A past LC-MS study (King et al., 2007) has shown that the HYNIC adduct contains fewer ligand co-ligand molecules, making it the most suitable chelator for 99mTc labelling as it can efficiently meet the ligand requirements of technetium. DTPA accomplishes the DPTA modification of peptides mainly by chelating NH2 bonds on the benzene ring and amino acids with COOH.111In radiolabelling them is an important labelling strategy for GLP-1R molecular imaging studies. When it comes to chelating agents, DOTA must occupy an important place. The four nitrogen atoms and the carboxyl group in the acetic acid on the nitrogen atom can be coordinated with metal, which is a common class of bifunctional chelators. 111In and 68Ga in GLP-1R molecular imaging can be perfectly targeted with it. It has also become one of the most widely used chelating agents in molecular imaging conversion. Others include NOTA, NODAGA and DO3A chelating agents are new chelating agents that have emerged in recent years. They and their derivatives have improved structural remodeling to give more stability to the target-labeled peptides. Thus they are also starting to emerge in GLP-1R molecular imaging.

In contrast to translational clinical chelators, preclinical studies have included the successful preparation of a variety of derivative chelators with 18F targeting markers. However, these chelators remain limited to preclinical studies, which may be related to the high cost of 18F radionuclides, high radiation burden, and cumbersome preparation of derivatives. A similar phenomenon is also seen in ligand selection. Both for Lys40-exendin-4 and Cys-exendin-4, their ligands are limited to exendin-4 itself. Therefore, there is an urgent need to find chelators with better affinity for the target peptide and to explore the preparation of more diverse and stable ligands in the future. This is also the key to successful diagnostic integration in GLP-1R molecular imaging.

## 5 Discussion

GLP-1R molecular imaging is currently the most promising non-invasive method for detecting benign insulinoma and other GLP-1R-positive diseases. The experimental expansion of various molecular probes has become a trend, and 68Ga-exendin has emerged in clinical transformation, becoming an efficient target probe choice for the localization of insulinoma. The diversity of nuclides in preclinical studies is awkward in the transition to clinical studies, as shown by our summary of the molecular imaging results of insulinoma. The main radiolabels that have been successfully translated into clinical practice are 111In, 99mTc and 68Ga. This is mainly due to their relatively low radiation load, the relative ease of preparation and their economic cost. The long half-life nuclides 89Zr, 64Cu and 177Lu, on the other hand, have greatly hindered the progress of their integration into clinical translational therapy due to the disadvantages of their high renal burden. This is the main issue that we need to urgently address in the future in the field of GLP-1R therapy.

Meanwhile, the choice of ligand is also crucial in determining the labelling strategy and the prerequisite basis for peptide preparation. It has been mentioned many times that current research, both pre-clinical and clinical, is focused around exendin-4 ligands. The ligand, although based on exendin-4 taps into a more established type of more diverse forms including Lys40-exendin-4 and Cys40-exendin-4. However, the strategy of exendin-4-based peptide synthesis and preparation has not yet left the core of exendin-4. Is it possible to explore the future from the new generation of GLP-1R analogues such as semiglutide and use them as substrates to develop more ligands and peptides with better stability and lower renal radiation? Such a direction may become a new phase of exploration for GLP-1R molecular imaging afterwards. On the one hand, the high renal uptake of exendin-4 limits its clinical application and translation. Although the preparation of different derivatives to reduce renal uptake is still troublesome. It may lead to tumors in the distal tail of the pancreas that overlap with the kidneys. As a result, these lesions are missed during imaging, and the average uptake of duodenal Brunner’s glands may also be a source of confusion for tumor imaging with this tracer (Zhu et al., 2017; Hepprich et al., 2020). On the other hand, the treatment of choice for insulinoma remains surgery, leading to limitations in molecular imaging of therapeutic nuclei. Therefore it becomes the biggest obstacle to advancing GLP-1R molecular medicine treatment in insulinoma.

In previous studies, adverse reactions such as nausea, vomiting, and hypoglycemia have been discovered in patients after imaging (C et al., 2020; Antwi et al., 2019; Shah et al., 2021). Therefore, the possibility to explore some new molecular probes targeting GLP-1R besides exendin is an uncharted area that we need to overcome and develop in the future. Clinical trials in NASH, pancreatic cancer, obesity, and cardiovascular disease are in full swing (Jansen et al., 2019). This article provides an overview of the current status of GLP-1R molecular imaging in insulinoma, endocrinology and other related fields (Table 6). It is clear that GLP-1R molecular imaging research continues to be centered on exendin. The search for novel molecular probes has become vital to advancing therapeutics in molecular medicine. The most recent GLP-1RA of the second generation is semaglutide. Its introduction and the success of clinical trials in several areas have prompted us to wonder whether this new GLP-1RA can be labeled with nuclides (Knudsen and Lau, 2019; Meier, 2021; Smits and Van Raalte, 2021). It has more homology than exendin-4 and may replace it as a new target tracer for GLP-1R molecular imaging. Nevertheless, this is only our advice, continue to speak with research data. Developing new molecular probes will result in significant advances in GLP-1R molecular imaging, which is exciting.

**TABLE 6:** Summary of GLP1R molecular imaging in other fields.

Year	Chelator	Compound	Preclinical/Clinical	Object	Significance	Deficiency	References
Koner 2007	None	exendin-4	clinical	GLP1R positive tumors	GLP1R is also highly expressed in gastrinoma, medullary thyroid carcinoma (MTC), 60% of pheochromocytoma	Lack of radionuclide labeling	Korner et al. (2007)
2018	Dotatate	68Ga-Dotatate-exendin-4	clinical	malignant insulinoma	Insulinomas with GLP1R negative expression are malignant and predominantly express SSTR	Small sample size	Bongetti et al. (2018)
2013	HYNIC	[Lys (40) (Ahx-HYNIC-(99m)Tc/EDDA)NH2]-Exendin-4	clinical	medullary thyroid carcinoma (MTC)	GLP1R imaging successfully detected thyroid tumors in 4 patients with MTC	Insufficient evidence for large sample sizes	Pach et al. (2013)
2012	FBA	18F-FBEM-Cys40-exendin-4	preclinical	myocardial ischemia/reperfusion (MI/R)	GLP1R imaging could dynamically monitor the dynamic changes of GLP-1R upregulation in the myocardial infarction/ischemia area	Myocardial cells from myocardial infarction have not been imaged and have not been clinically translated	Gao et al. (2012)
2020	NODAGA	68Ga-NODAGA-exendin-4	preclinical	myocardial infarction model	Dynamic imaging of 68Ga-NODAGA-exendin-4 in myocardial cells after myocardial infarction for the first time	Further clinical conversion of GLP1R molecular imaging in patients with myocardial infarction myocardial protection efficacy monitoring to create a new means	Stahle et al. (2020)
2008, 2011	None	Exendin-4	preclinical	PD model	exendin-4 can restore the nerve in the subventricular zone and hippocampus, thereby improving mice’s cognitive, motor, and memory abilities	The absence of nuclide-directed molecular probes has hindered the development of GLP1R imaging in these diseases	Bertilsson et al. (2008), Isacson et al. (2011)
2021	None	Exendin-4	preclinical	PD model	exendin-4 treatment attenuated the pathological accumulation of α-synuclein in PD	The absence of nuclide-directed molecular probes has hindered the development of GLP1R imaging in these diseases	Bu et al. (2021)
2020, 2021, 2022	None	exendin-4/DA-JC4/DA5-CH	preclinical	PD model	(Exendin-4 and DA-JC4) and GLP-1/GIP dual receptor agonists DA5-CH can successfully cross the blood-brain barrier and reach the pathological area of PD		Salameh et al. (2020), Lv et al. (2021), Zhang et al. (2022)
2018	NOTA	[18F]AlF-NOTA-MAL-Cys39-exendin-4	preclinical	neurodegenerative diseases	GLP1R expression in the brain of aged mice was elevated reduced	GLP1R receptor expression was explored only in animal models stratified by age, No neurodegenerative disease modeling and imaging research	Wang et al. (2018)
2020	FBA	[18F]FBEM-Cys 39-exendin-4	preclinical	AD model	The mechanism of 18F-exendin-4 penetrating blood-brain barrier and the decrease of GLP 1R expression in AD animal model were studied for the first time	Only comparisons between 18F-labeled derivatives have been performed, but further studies of GLP1R expression in AD models with different radionuclides are lacking	Liu et al. (2020)
2020	NODAGA	68Ga-NODAGA-exendin-4	clinical	Obesity	Detecting the difference in GLP1R intracranial distribution of obese patients compared with ordinary people	This tracer was not suitable for GLP1R distribution imaging in the brain of these patients	Deden et al. (2021)

The performance of GLP-1R molecular imaging in diabetic BCM gives us insight and hope for theranostic. Recent studies have discovered novel targets in neuroendocrine tumors, such as the glucose-dependent insulin-biophilic polypeptide receptor (GIPR), the same hormone receptor as GLP-1R. Because it is expressed in gastrinoma and bronchial lung cancer, it holds great promise for molecular imaging of NENs when the GLP-1R is negative (Papaefthymiou et al., 2022). The gallbladder hormone-2 (CCK2) receptor is overexpressed in MTC, small cell lung cancer, and other neuroendocrine tumors (Refardt et al., 2021) and is considered a promising new target for NENs therapy. Thus, our outlook and goal have been to develop novel peptide hormone receptors, apply novel GLP-1R drugs as molecular probes, and target GLP-1R diseases. These are emerging opportunities to open up gaps, even if the molecular medicine theranostic is challenging tomorrow.

## Abbreviations

GLP-1, Glucagon-like peptide-1; GLP-1R, Glucagon-like peptide-1 receptor; GLP-1R, Glucagon-like peptide-1 receptor agonists; DPP-IV, dipeptidyl peptidase IV; MTC, medullary thyroid carcinoma; EHH, endogenous hyperinsulinemic hypoglycemia; DTPA, Diethylene Triamine Pentaacetic Acid; HPLC, High Performance Liquid Chromatography; SSTR, somatostatin receptor; DOTATATE, DOTA-octreotide; EHCO, Echocardiogram; CAG, coronary angiography; CTCA, computed tomography coronary angiography; MRI, Magnetic Resonance Imaging; MI/R, myocardial ischemia/reperfusion; T2DM, type 2 diabetes mellitus; NASH, nonalcoholic steatohepatitis; AD, Alzheimer Disease; PD, Parkinson Diseaseto; BCM, beta (β)-cell mass; BB-DP, bio breeding diabetes-prone; CHI, Congenital Hyperinsulinemia; PGBH, post-gastric bypass hypoglycemic syndrome; NIPHS, non-insulinoma pancreatic hypoglycemic syndrome; MEN-1, Multiple endocrine neoplasia type 1; GIPR, glucose-dependent insulin-biophilic polypeptide receptor; CCK2, gallbladder hormone-2.
